# Comprehensive analysis of HSF genes from celery (*Apium graveolens* L.) and functional characterization of *AgHSFa6-1* in response to heat stress

**DOI:** 10.3389/fpls.2023.1132307

**Published:** 2023-05-08

**Authors:** Mengyao Li, Ran Zhang, Jin Zhou, Jiageng Du, Xiaoyan Li, Yong Zhang, Qing Chen, Yan Wang, Yuanxiu Lin, Yunting Zhang, Wen He, Xiaorong Wang, Aisheng Xiong, Ya Luo, Haoru Tang

**Affiliations:** ^1^ College of Horticulture, Sichuan Agricultural University, Chengdu, China; ^2^ Institute of Pomology and Olericulture, Sichuan Agricultural University, Chengdu, China; ^3^ College of Horticulture, Nanjing Agricultural University, Nanjing, China

**Keywords:** celery, heat shock transcription factor, heat stress, function annotation, transgenic, transcriptional regulation

## Abstract

High temperature stress is regarded as one of the significant abiotic stresses affecting the composition and distribution of natural habitats and the productivity of agriculturally significant plants worldwide. The HSF family is one of the most important transcription factors (TFs) families in plants and capable of responding rapidly to heat and other abiotic stresses. In this study, 29 *AgHSFs* were identified in celery and classified into three classes (A, B, and C) and 14 subgroups. The gene structures of *AgHSFs* in same subgroups were conserved, whereas in different classes were varied. AgHSF proteins were predicted to be involved in multiple biological processes by interacting with other proteins. Expression analysis revealed that *AgHSF* genes play a significant role in response to heat stress. Subsequently, *AgHSFa6-1*, which was significantly induced by high temperature, was selected for functional validation. *AgHSFa6-1* was identified as a nuclear protein, and can upregulate the expression of certain downstream genes (*HSP98.7*, *HSP70-1*, *BOB1*, *CPN60B*, *ADH2*, *APX1*, *GOLS1*) in response to high-temperature treatment. Overexpression of *AgHSFa6-1* in yeast and *Arabidopsis* displayed higher thermotolerance, both morphologically and physiologically. In response to heat stress, the transgenic plants produced considerably more proline, solute protein, antioxidant enzymes, and less MDA than wild-type (WT) plants. Overall, this study revealed that *AgHSF* family members perform a key role in response to high temperature, and *AgHSFa6-1* acts as a positive regulator by augmenting the ROS-scavenging system to maintain membrane integrity, reducing stomatal apertures to control water loss, and upregulating the expression level of heat-stress sensitive genes to improve celery thermotolerance.

## 1 Introduction

Celery (*Apium graveolens* L.) is a valuable edible and medical vegetable crop belonging to the Apiaceae family that originated in the Mediterranean and the Middle East and is now extensively cultivated and consumed worldwide (Li et al., 2014; Li et al., 2018). However, celery cannot endure high temperatures; its optimal germination and growth temperatures are 15-20 °C and 15-25 °C, respectively (Richard, 2004). Because of this, heat stress negatively affects celery growth and yield. Increasing the heat tolerance of celery can materialize its annual supply and significantly improve its quality and production. Plants have developed a variety of defense strategies to relieve abiotic stresses, including morphological mechanisms (Zhao et al., 2022), plant hormone regulation (Waadt et al., 2022), and modulating the expression of stress-responsive genes (Zhou et al., 2019). Previous studies have shown that some transcription factors (TFs), such as bZIP, DREB, MYB and HSF, are the key regulators of several horticultural plants response to various abiotic and biotic stress (Kumar et al., 2021; Li et al., 2022b).

Heat shock transcription factors (HSFs) are one of the plant’s most important transcription factors (Fan et al., 2021). The HSF family members can respond to heat stress and other abiotic stresses, including cold, salt, and drought, by binding to the reverse repeat region of heat shock elements (HSEs) and promoting transcription of heat shock proteins (HSPs) (Guo et al., 2016; Jacob et al., 2017). Although the members of the HSF family have different functions, their structures are highly similar (Chen et al., 2018). Typically, plant HSF protein has a conserved patterned structure, including a DNA-binding domain (DBD) at the N-terminus, two hydrophobic 7-peptide repeats (HR-A/B region) oligomerization domain (OD), nuclear localization signal (NLS), nuclear export signal (NES), activation structure (AHA motif), and repressor domain (RD) (Guo et al., 2016; Jiang et al., 2021). The HSF family in plants has been further classified into three classes (HSFA, HSFB, and HSFC) based on the length of their basic amino acid (aa) sequences between the DBD and HR-A/B regions and the number of amino acid residues inserted into the HR-A/B regions (Nover et al., 2001; Liu et al., 2018).

The study demonstrated that HSFA members played a primary role in responding to heat stress, while HSFB assists HSFA in its response to heat (Chan-Schaminet et al., 2009). Moreover, study has found that the HSFA genes tends to regulate the expression of downstream related HSP genes to improve plant thermotolerance, while the HSFB genes negatively regulates the expression of HSP genes (Wang et al., 2021). In recent years, several studies have supported that HSFA genes exhibit similar role in heat stress. For example, overexpression of *AtHsfA1a* in *Arabidopsis* can enhance heat stress tolerance in transgenic plants (Qian et al., 2014). The overexpression of maize *ZmHsfA2* in the *Arabidopsis hsfa2* mutant restored heat tolerance and raised the survival rate after heat stress (Li et al., 2019). However, much less is known about the function of HSFB and HSFC genes. It is demonstrated that HSFB inhabited the growth of roots and aerial organs in transgenic *Arabidopsis*, and could help plants acquire thermotolerance (Tan et al., 2021). The studies presented above suggest that some HSFs played critical roles in enhancing heat tolerance in plant.

Although HSF transcription factors have been previously found in celery, this study only identified 17 HSF genes and also did not determine their functions (Li et al., 2018). The response network studies on HSF in celery are still limited. In the present study, 29 *AgHSF* genes were identified in celery, and *AgHSFa6-1* was isolated for further investigation of its activities under heat stress by transferring into *Saccharomyces cerevisiae* and *Arabidopsis thaliana*. Transgenic lines displayed higher thermotolerance than wild-type (WT)in morphologically and physiologically, as shown by increased antioxidant enzyme activity, proline content, solute protein, etc. These results revealed that overexpression of *AgHSFa6-1* enhanced the tolerance of transgenic plants mainly through boosting the ROS-scavenging system, osmoregulation, and a specific molecular mechanism. In conclusion, this study established a response network of *AgHSFa6-1* in maintaining heat tolerance, which provide a theoretical basis for celery breeding and cultivating.

## 2 Materials and methods

### 2.1 Identification of HSF family in celery and phylogenetic relationship analysis

Genome sequences were downloaded from published celery genome database (http://apiaceae.njau.edu.cn/celerydb and http://celerydb.bio2db.com/) for constructing the local BLAST (Li et al., 2022b). The hidden Markov model profile of the HSF domain (PF00447) from the Pfam database (http://pfam.xfam.org/) was downloaded and utilized to identify the HSF members in celery using the HMMER 3.0 program with default parameters. Candidate proteins were further submitted to NCBI-CDD (https://www.ncbi.nlm.nih.gov/cdd) to confirm the conserved domains. The physicochemical properties of the HSF genes identified from celery were analyzed using online ExPASy software (http://web.expasy.org/protparam). The subcellular localization was predicted with WOLF PSORT (https://www.genscript.com/tools/wolf-psort) and SoftberryProtComp 9.0 (http://www.softberry.com). The HSF sequences of *Arabidopsis* were downloaded from TAIR (https://www.arabidopsis.org/). ClustalX (v2.1) was used to align multiple sequences of all HSFs with default parameters, and phylogenetic tree was constructed by MEGA7.0 using neighbor-joining (NJ) method with 1000 bootstrap replicates.

### 2.2 Chromosomal distribution and gene structure, and conserved motif analysis of AgHSFs

The chromosomal location of *AgHSFs* was visualized through TBtools software (Chen et al., 2020). The exon/intron structure of *AgHSFs* was analyzed by online tool Gene Structure Displayer Server v2.0 (http://gsds.gao-lab.org/). Conserved motifs were analyzed using MEME Suite v5.4.1 with the parameters set as follows: the maximum number of motifs, 20; and the optimum width of each motif, between 10 and 50 residues.

### 2.3 Promoter region and interaction network analysis

The *cis*-regulatory elements in the 1.5 kb promoter regions of *AgHSFs* were predicted using the PlantCARE database (http://bioinformatics.psb.ugent.be/webtools/plantcare/html) (Lescot et al., 2002). The STRING software was used to output the protein interaction value data between *AgHSFs* and other proteins in celery genome, and established protein interaction network. Results were visualized with Cytoscape software v3.7 (Shannon et al., 2003).

### 2.4 Gene cloning and subcellular localization

The heat-tolerant variety ‘Jinnan Shiqin’ and the non-heat-tolerant variety ‘Liuhe Huangxinqin’ were used as plant materials. Celery seeds were placed in an incubator at 20 °C in dark for germination, and then transferred them into growth chambers at 25 °C/20 °C (14 h light/10 h dark cycles). When the plants reached the stage of about 10 leaves, they were transferred into 38 °C for 24 h heat stress. The celery leave samples were collected after at 0 h (QC1), 4 h (QC2), 12 h (QC3), and 24 h (QC4) of treatment. Plant RNA samples were extracted using a total RNA kit (Tiangen, Beijing, China), and then converted into cDNA using PrimeScript RT Kit (TaKaRa, Dalian, China). The sequences of *AgHSF* genes were amplified for RT-PCR using *EasyTaq* DNA Polymerase Kit (TransGen Biotech, Beijing, China). The PCR product was ligated with *pEASY*-T1 vector and sequenced in Tsingke Biotechnology Co., Ltd. (Beijing, China).

The full-length of *AgHSFa6-1* was amplified using specific primers (**Table S1**), and inserted into green fluorescent protein (GFP)-fusion expression vector pA7. The expression vector *35S*:*AgHSFa6-1*:*GFP* was constructed with two *Bam*H I restriction enzyme sites. The empty vector containing 35S::GFP protein served as a control. The fusion expression and empty vector were transformed in tobacco leaves by DNA particle bombardment system (PDS-1000, Bio-Rad, USA). The tobacco was firstly disposed in dark for 24 h, fluorescence was then observed using a laser confocal microscope (LSM800, Zeiss, Germany).

### 2.5 Transformation in yeast and Arabidopsis and stress tolerance analysis

The full-length open reading fragments of *AgHSFa6-1* were amplified by PCR using specific primers, and inserted into the pYES2 vector (**Table S1**). Then the recombinant vector was transformed into *Saccharomyces cerevisiae* strain *INVSC1*, and positive transformants were selected in the SD-Ura medium at 30 °C. For heat stress treatment, the positive yeasts were cultured on the SG-Ura medium for 3 d at 30 °C, 39 °C, 41 °C and 45 °C, respectively.

For the overexpression of *AgHSFa6-1*, the double-cauliflower mosaic virus 35S (CaMV 35S) promoter and NOS terminator from pSAT1-cEYFP-N1 were amplified and subcloned into the vector pCAMBIA1301. To obtain transgenic *Arabidopsis*, the amplification product was inserted downstream of the double CaMV 35S promoter into the modified pCAMBIA1301 vector between *BamH* I and *Sac* I sites. The construct contains the CaMV 35S promoter driving *AgHSFa6-1* and the CaMV 35S promoter driving the *Hyg* gene for hygromycin resistance as a selectable marker. The pCAMBIA1301 vector also contained a GUS reporter gene following CaMV 35S promoter. The recombinant vector was introduced into the *Agrobacterium tumefaciens* strain GV3101 through electroporation. Then, it was transformed into *Arabidopsis* Columbia (Col-0) wild-type (WT) plants by floral-dip method (Zhang et al., 2006). The transgenic T_0_ lines were selected using hygromycin on MS medium, and the hygromycin-resistant plants were selected out to obtain T_1_ seeds for PCR assays. Then, consistently selected twice to harvest T_2_ seeds of transgenic *Arabidopsis*, and the T_3_ homozygous lines were used for further verification.

Seeds of *Arabidopsis* were placed on 1/2 MS medium to germination. The seeds were vernalized in a dark incubator at 4 °C for three days and then changed the growth condition at 25 °C/15 °C (16 h light/8 h dark cycles) and 70% relative humidity. Ten-day-old seedlings were subjected to heat stress for three days at 38 °C/25 °C (16 h light/8 h dark cycles), and then measured the primary root length. Two-month-old seedlings were subjected to 38 °C/25 °C heat stress for 24 h. They were sampled at 0 h, 4 h, 12 h, and 24 h for RNA extraction utilized for subsequent RT-qPCR analysis. Moreover, leaf-samples of 4 h were collected for stomata analysis and samples of 24 h were collected for physiological measurements. Then plants were domesticated at 42 °C for two days to compare their phenotypic difference.

### 2.6 Measurements of physiological parameters

The free proline content was measured according to the acidic-ninhydrin-based colorimetric method (Bates et al., 1973). The activities of superoxide dismutase (SOD) and peroxidase (POD) were measured using the nitrogen blue tetrazolium (NBT) and the guaiacol methods, respectively (Macadam et al., 1992; Cao et al., 2018). The concentration of malondialdehyde (MDA) in leaves was measured by the TBA colorimetric method (Li et al., 2020). For stomata analysis, the plant samples were immersed into FAA fix solution, dehydrated, embedded in paraffin, sectioned, and stained with toluidine blue. The width and length of the stomatal aperture were analyzed using ImageJ software v1.8.0.112 (Graphics software; NationalInstitutes of Health: Wayne Rasband, USA, 1997).

During above experiment, three independent replicates of each transgenic lines were performed. All of the data were analyzed using SPSS 26.0 software (SPSS Inc., Chicago, IL, USA), and the significance levels were based on P < 0.05 (*) and P < 0.01 (**).

### 2.7 Gene expression and reverse transcription-quantitative PCR (RT-qPCR) analysis

The transcriptome data of celery under variable high temperature was obtained by this research group (Li et al., 2022a), and the transcript abundance of *AgHSF* was calculated in log2(FPKM) to generated cluster heatmaps.

RT-qPCR was performed using SYBR Premix Ex Taq (TaKaRa, Dalian, China). All the steps were followed the manufacturer’s instruction (CFx384TM Real-Time System, Bio-Rad, USA), and the expression level was calculated by the 2^-△△Ct^ method (Pfaffla, 2001). *AgTUB* and *AtACT2* were served as reference genes for celery and *Arabidopsis* genes, respectively. All gene primer sequences used in this study were designed using Primer Premier software (version 6.0; Premier Biosoft International: Palo Alto, CA), and the sequences are listed in **Table S1**.

## 3 Results

### 3.1 Identification of HSF family genes in celery and phylogenetic analysis

A total of 29 *AgHSF* genes were identified and re-named from *AgHSFa6-1* to *AgHSFc1-1* (**Table S2**). The results demonstrated that almost all the genes were localized in the nucleus. The polypeptides of AgHSF proteins ranged from 232 (AgHSFa9-2)-503 aa (AgHSFa1-3), with the molecular weight ranging from 26450.87 (AgHSFa9-2)-55586.82 Da (AgHSFa1-3). The aliphatic amino acids accounted for 18%, whereas the aromatic amino acids accounted for only 8%. The theoretical isoelectric point (*pI*) of proteins belongs to class A ranged between 4.83-6.25, indicating that they were acidic. However, except for B2, the *pI* values of class B proteins were more than 7, suggesting they were alkaline. All AgHSF proteins had negative GRAVY values, which means that all proteins were hydrophilic.

**Figure 1:**
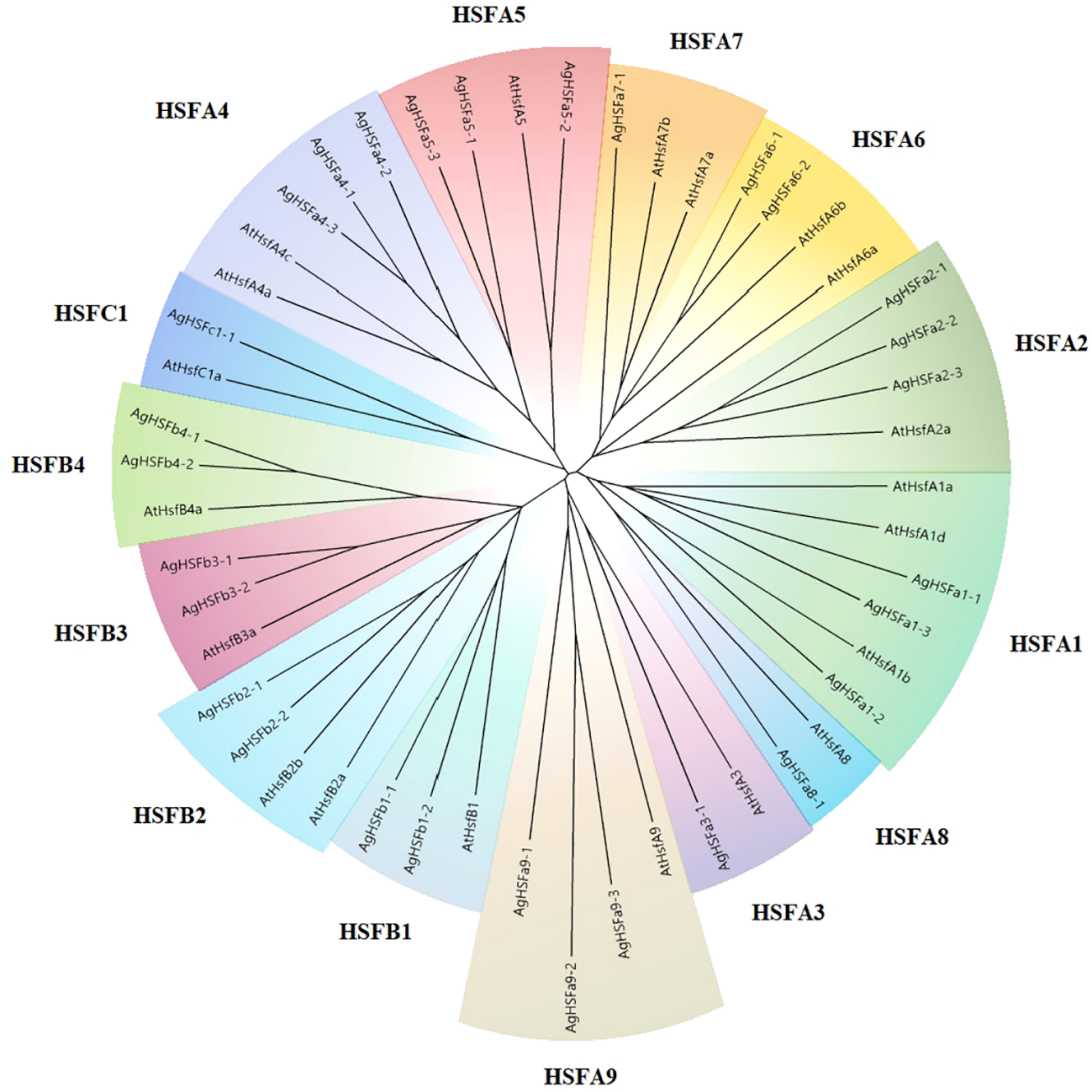
The phylogenetic tree of the HSF proteins in celery and *Arabidopsis*. The phylogenetic tree was generated using the NJ method with 1000 bootstrap replicates. Different colors and letters represent various subgroups.

A phylogenetic tree was created between 29 celery and 20 *Arabidopsis* HSF proteins to determine their categorization and explore their evolutionary relationship (**Figure 1**). The result revealed that the relationships of HSF genes between the two species were highly homologous. The AgHSFs in celery were classified into three classes and 14 subgroups. The class A had the most abundant proteins, which were clustered into nine subgroups, corresponding to HSFA1-HSFA9, and class B were clustered into four subgroups, corresponding to HSFB1-HSFB4. In contrast, the class C only had one subgroup for one gene, corresponding to HSFC1.

### 3.2 Gene structure analysis

**Figure 2:**
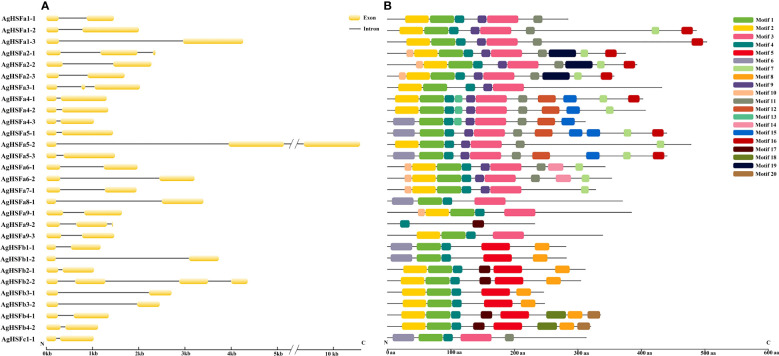
Analysis of gene structure and conserved motifs of *AgHSF* genes. **(A)** The exon and intron structure of *AgHSF* genes. The nucleotide sequence of these genes was used for analysis, and box represented exons and line represented introns. **(B)** Display of conserved motifs in AgHSF proteins. The protein sequences were subjected to Motif Search based on homology, and boxes represented motifs and different colors represent different types of motifs.

The evolution of gene family members was mainly determined by the variation of their conserved motifs and the diversity of gene structures (Hu et al., 2010). Results revealed that the longest intron was nearly 4 Kb (*AgHSFa5-2*), and all *AgHSF* genes had between two and four exons and one to three introns (**Figure 2A**). However, the types and numbers of motifs varied considerably (**Figure 2B**). A total of 20 motifs were detected in AgHSF proteins; the sequence of each motif is listed in the supplementary table (**Table S3**). All 29 AgHSFs shared only one motif type (motif 4), and the number of motifs varied from two to 11. Similarities were more prevalent among the same groups or classes. AgHSFs belongs to Class A usually had more motif types, and except for AgHSFa9-2, they had motifs 1 and 3 in common, and members belongs to class B had motifs 1, 4, 5, and 8 in common. Moreover, motifs were always arranged in a particular order, such as motifs 2- 1- 4- 3 and 9. The highly conserved exon-intron distribution and motif divergence indicated that AgHSFs evolved according to a group, establishing the basis for the functional diversity of different classes.

### 3.3 Chromosomal distribution and cis-regulatory elements of AgHSFs promoter regions

**Figure 3:**
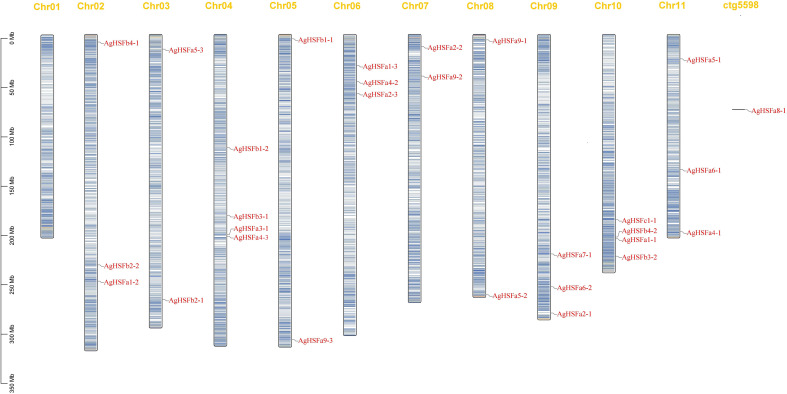
Chromosome location of *AgHSF* genes. The bars represented chromosomes, and chromosome numbers was indicated at the top of each bar with length marked at the left of whole figure.

To identify the localization of *AgHSF* genes, the TBtools software was used to visualize the location of these genes on different chromosomes in the celery genome (**Figure 3**). Result demonstrated that except for Chr01, 28 *AgHSF* genes were unevenly distributed between Chr02 and Chr11, whereas *AgHSFa8-1* was located at an unanchored contig. The Chr04 and Chr10 had the largest number of genes (four genes), followed by Chr02, 06, 09, and 11 with three genes each. Genes belongs to class A were located on each chromosome from Chr02 to 11, while the class B genes were only present on Chr02, 03, 04, 05, and 10, and the C class genes were only located on Chr10. Some *AgHSF* genes demonstrated high cross collinearity, clustering at similar sites on the same chromosome. For instance, *AgHSFa3-1* and *AgHSFa4-3* were localized at a similar site on the same chromosome, revealing that they might have sequence similarity.

**Figure 4:**
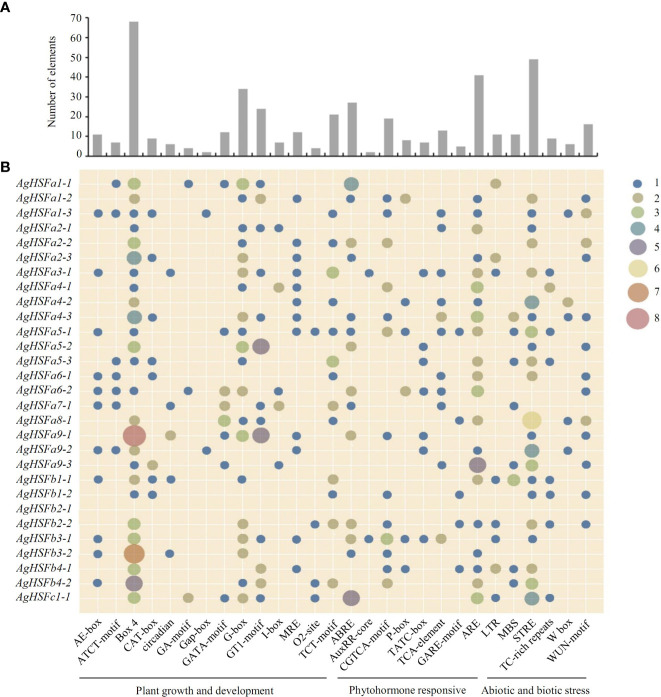
Number and distribution of cis-regulatory elements in the promoter region of *AgHSF* genes. **(A)** The total number of each element. **(B)** The distribution of elements in 29 *AgHSFs* promoter regions. The numbers (from 1 to 8) of each element were shown as different types of dots, and the bigger dot meant bigger number.

Transcription factors can regulate gene expression by binding to cis-regulatory elements in response to different biotic or abiotic stress signals (Li et al., 2021). Various cis-regulatory elements were predicted in promoter sequences of all the *AgHSF* genes, and they were divided into three groups based on their functions: plant growth and development, phytohormone responsive, and abiotic and biotic stress (**Figure 4B**). Result revealed that some elements were widely distributed in promoter regions of *AgHSFs*, with light response elements (Box 4, G-box, and GT1-motif) being the most frequently distributed, followed by antioxidant response elements (ARE) and stress-response element (STRE). Furthermore, abscisic acid response elements (ABRE) were also detected in promoter regions of several *AgHSFs* (**Figure 4A**), indicating these genes can regulate the abscisic acid (ABA) secretion or synthesis process in response to heat shock.

### 3.4 Protein interaction network analysis of AgHSFs proteins

**Figure 5:**
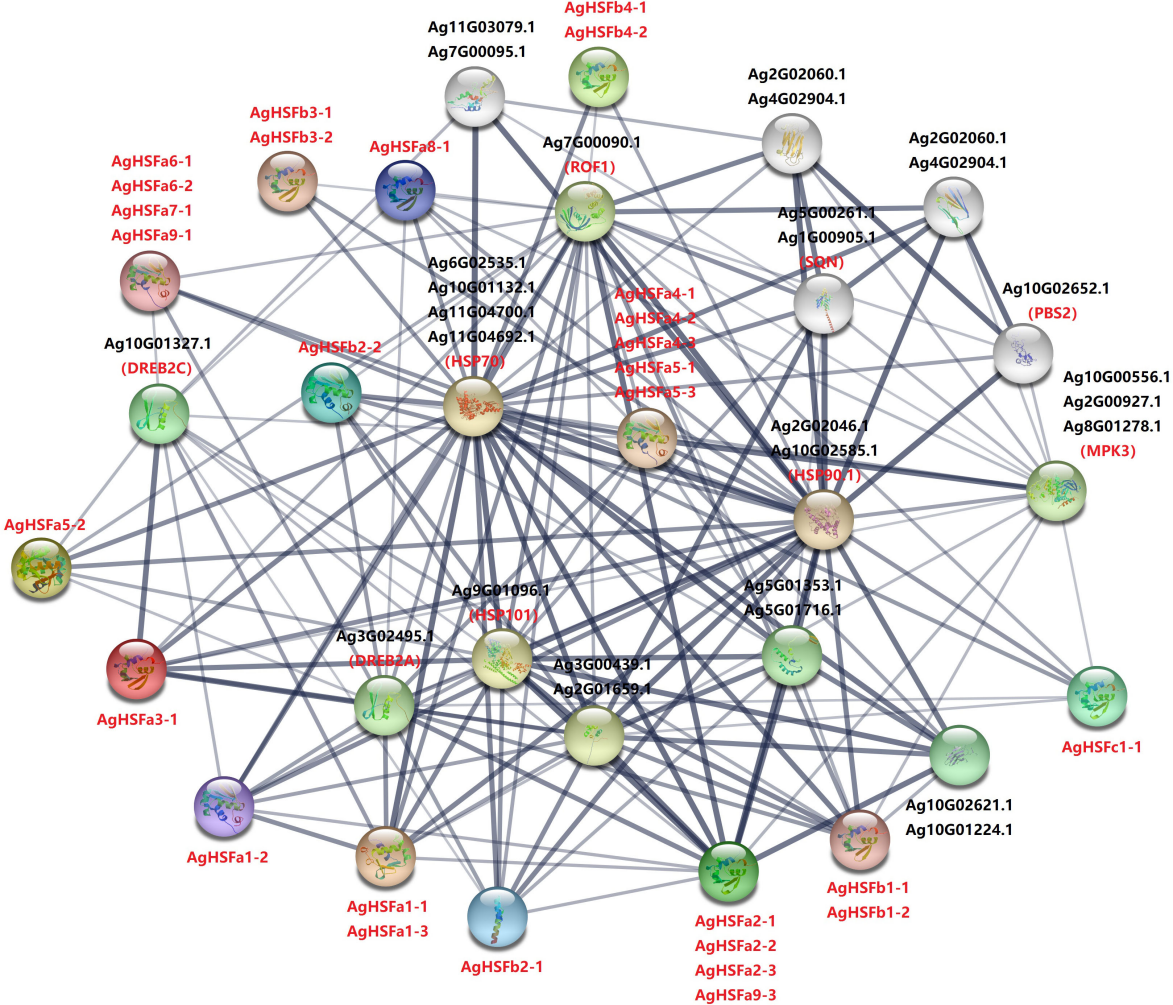
Interaction network of AgHSF in celery according to the orthologs in *Arabidopsis*. Every circle represents different protein, and all the letters are the name of proteins. The width and shade of lines represent the correlation strength of each proteins.

To further investigate the regulatory mechanism of HSF genes in celery, a protein interaction network comprising the homologous proteins in *Arabidopsis* was constructed (**Figure 5**). Except for AgHSFa9-2, 28 AgHSF proteins and 15 other proteins in the celery genome were present in this interaction network. Out of these, three HSP proteins constituted the central node and were connected with multiple AgHSF proteins. AgHSF proteins also exhibited significant relationships with DREB, PBS, MPK, and SQN proteins. HSF genes were proved to respond to heat shock by regulating the expression of HSP (Jacob et al., 2017). In our study, members of HSF family and HSP formed an expansive regulation network, indicating that these genes may play important roles in sensing and responding to heat stress.

### 3.5 Transcript abundance and expression pattern analysis of AgHSFs under heat stress treatments

**Figure 6:**
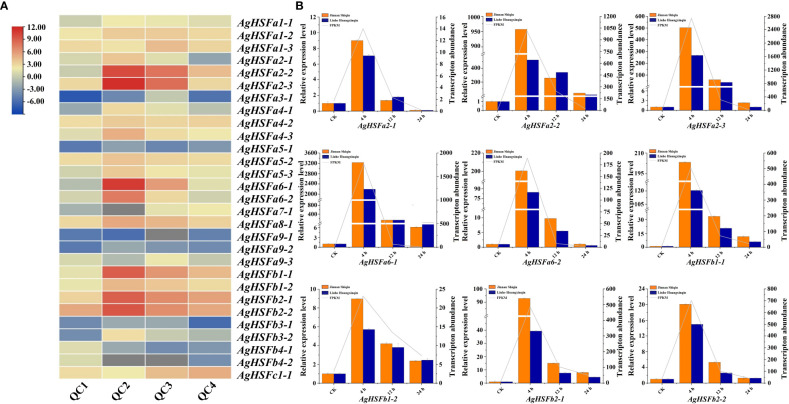
Expression pattern and expression level analysis of *AgHSF* genes. **(A)** Expression pattern of 29 *AgHSFs* under heat stress treatment based on RNA-seq data. QC1, QC2, QC3, and QC4 represented the samples treated at high temperature for 0 h, 4 h, 12 h, and 24 h, respectively. The expression values were normalized to reads per kilobase per million mapped reads (FPKM), and the data was calculated using log2 (FPKM). **(B)** Expression level analysis of nine highly expressed *AgHSFs*. Different colors represented different celery varieties, and line represented transcription abundance.

According to the transcriptome data, all the 29 *AgHSF* genes were expressed after 0 h, 4h, 12 h, and 24 h of heat stress, referring to QC1, QC2, QC3, and QC4, respectively. Previous studies demonstrated that *HSFA2* genes in *Arabidopsis* and tomato could be induced by heat stress, resulting in enhanced permeability and antioxidant capacity of plants (Meiri et al., 2010; Fragkostefanakis et al., 2016). Several *AgHSFs*, including *AgHSFa2-2*, *AgHSFa2-3*, and *AgHSFa6-1*, displayed a typical trend of initially increasing and declining. The expression levels of *AgHSFa6-1* increased significantly at QC2 and reached their peak at this stage but decreased during QC3 and QC4 (**Figure 6A**). Despite this, their expression levels were significantly higher than any other *AgHSF* genes.

Nine highly expressed *AgHSFs* (*AgHSFa2-1*, *AgHSFa2-2*, *AgHSFa2-3*, *AgHSFa6-1*, *AgHSFa6-2*, *AgHSFb1-1*, *AgHSFb1-2*, *AgHSFb2-1*, *AgHSFb2-2*) were further detected by RT-qPCR to validate their expression levels in two celery cultivars (heat-tolerant celery variety ‘Jinnan Shiqin’ and the non-heat-tolerant celery variety ‘Liuhe Huangxinqin’) (**Figure 6B**). The expression levels of nine *AgHSFs* were all exhibited higher expression level in ‘Jinnan Shiqin’ after 4, 12, 24 h heat stress, especially *AgHSFa6-1* exhibited a thousand times higher expression level than CK after 4 h heat stress. This result revealed that *AgHSFa6-1* were involved in the response to heat stress, and the heat-tolerant celery variety could response to heat stress more rapidly to adapt to high temperature. Overall, similar expression trends in gene expression were detected from transcriptome data and RT-qPCR analyses.

### 3.6 Gene clone and subcellular localization of AgHSFa6-1

**Figure 7:**
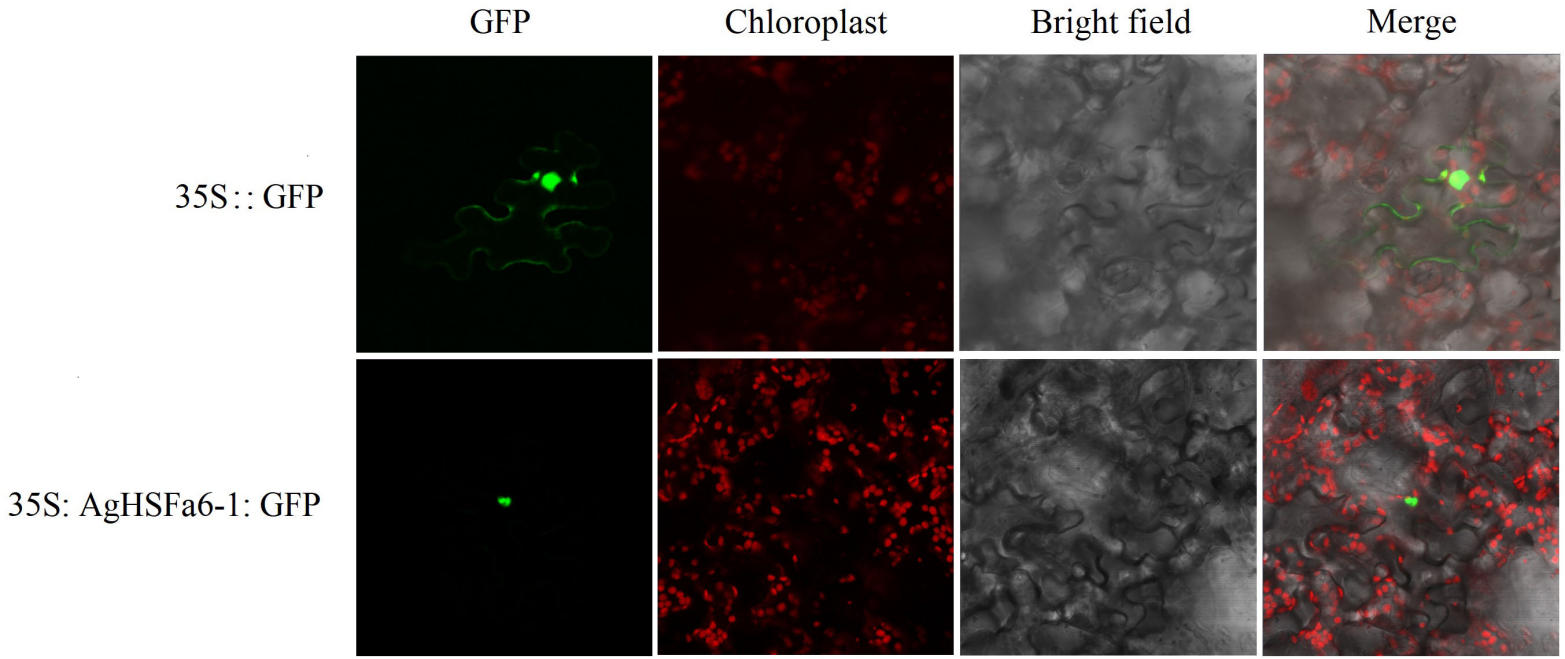
Subcellular localization of AgHSFa6-1 proteins in tobacco cells. Green signal represented the localization of AgHSFa6-1proteins.

Result demonstrated that the control was strongly expressed in the cytomembrane and nucleus, whereas 35S:AgHSFa6-1:GFP was detected only in the nucleus, indicating that the AgHSFa6-1 protein was targeted in the nucleus (**Figure 7**). The result was consistent with previous subcellular localization predictions based on protein structures and demonstrated that AgHSFa6-1 could execute the function of a transcription factor.

### 3.7 Analysis of AgHSFa6-1 overexpression in yeast and A. thaliana

**Figure 8:**
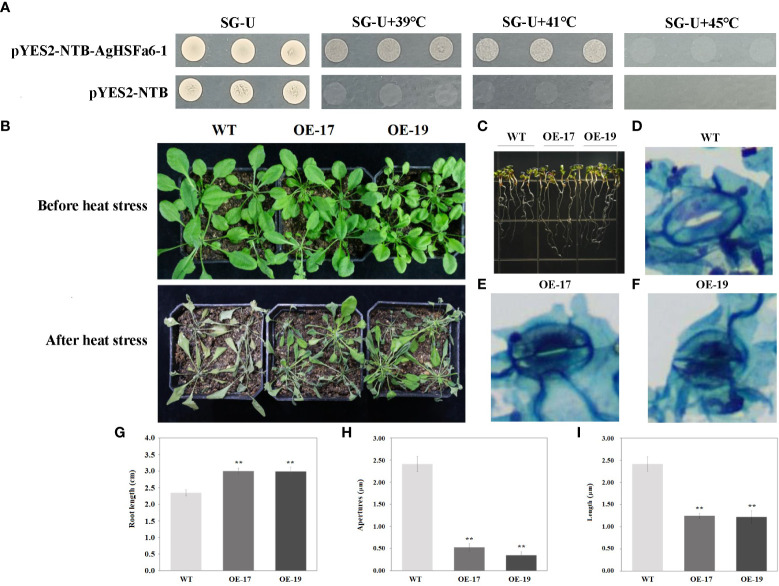
The transgenic results of overexpression *AgHSFa6-1* in yeast and *Arabidopsis*. **(A)** The growth pattern of control strain (pYES2-NBT) and recombinant strain (pYES2-NBT-AgHSFa6-1) on SG-Ura medium. The yeasts grew at different temperatures for 3 **(D) (B)** Morphological differences between transgenic and WT plants under heat stress. **(C, G)** Root-length differences between transgenic and WT plants. **(D–F, H, I)** Electron microscope photos of stomata after heat stress, and statistics of stomata apertures and length. Average root-length, stomata apertures and length of transgenic and WT plants analyzed by SPSS 26.0. ** represented the differences were significant.

The yeasts were cultured on SG-Ura medium at 30, 39, 41, and 45 °C for three days (**Figure 8A**). Result demonstrated that the control strain only grew at 30 °C, while the transgenic strains grew significantly at 39 and 41 °C. However, at the temperature of 45 °C, neither the control nor transgenic strains grew, suggesting that the *AgHSFa6-1* gene responds to temperature stresses in yeast.

The transgenic *Arabidopsis* plants were obtained using hygromycin selection, and their validity was verified by PCR and GUS staining (**Figure S1**). A total of 25 transgenic lines were obtained and designated as from OE-1 to OE-25. Two lines of transgenic lines (OE-17 and OE-19) and WT plants were selected to verify the *AgHSFa6-1* function in response to heat stress. The morphological and root-length differences were measured using ten-days-old and two-month-old seedlings. As illustrated in **Figures 8B, C**, under normal growth conditions, OE-17, OE-19, and WT grew consistently, whereas after 24 h of continuous heat stress at 38/25 °C, the WT plants visibly withered to death, but the transgenic seedlings were still alive. Additionally, the growth of WT seedlings was stalled by heat stress, whereas OE-17 and OE-19 seedlings survived, and the average root length of transgenic plants was significantly longer than that of WT (**Figures 8C, G**).

Stomata are the main channels for gas exchange, and plants normally adjust the stomatal conductance to vary their temperature (Wang et al., 2022). When exposed to 4 h of heat stress, the conductance of WT plants was more open than that of transgenic plants, and both the aperture size and length of WT stomata were significantly larger than those of transgenic stomata (**Figures 8D–F, H, I**). These findings demonstrated that over-expression *AgHSFa6-1* enhanced the heat-endurance ability of transgenic plants by regulating stomatal conductance and opening to control water loss.

### 3.8 Physiological and gene expression analysis of transgenic and wild-type A. thaliana plants under heat stress

**Figure 9:**
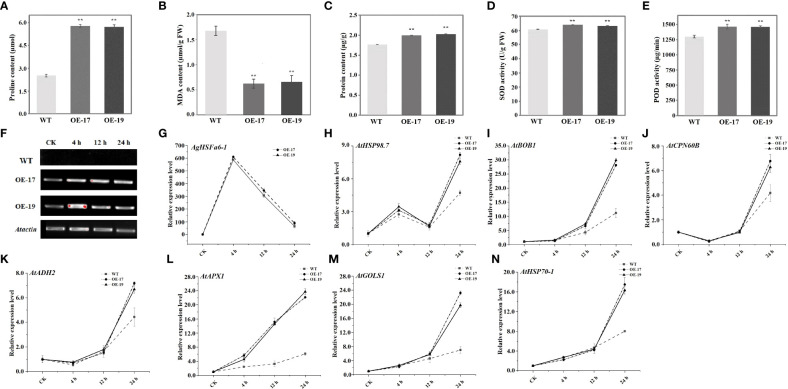
Analysis of physiological traits in transgenic and WT plants and expression profiles of heat-related genes under heat stress treatment. ** represented the differences were significant. **(A)** Proline contents in two transgenic and WT plants measured after 24 h heat stress. **(B)** MDA contents in in two transgenic and WT plants measured after 24 h heat stress. **(C)** Soluble protein contents in two transgenic and WT plants measured after 24 h heat stress. **(D, E)** Activity of SOD and POD enzyme in in two transgenic and WT plants measured after 24 h heat stress. **(F, G)** The expression levels of *AgHSFa6-1* in WT, OE-17 and OE-19. **(H–N)** The expression levels of stress-responsive genes in WT, OE-17 and OE-19. Vertical axis represented expression levels of each genes, and horizontal axis represented stages (CK was control).

Leaf samples from two-month-old *Arabidopsis* plants were used to measure all physiological parameters. The physiological indices of WT and transgenic plants under heat stress were determined, including proline, MDA, soluble protein, and antioxidant enzymes (SOD and POD). Results demonstrated that the content of proline in transgenic plants were significantly higher than in WT plants, while the MDA content displayed the opposite trend (**Figures 9A, B**). Proline is a signal molecule that regulates osmotic pressure, and MDA reflects the degree of membrane damage (Liu et al., 2016; Fu et al., 2018). Examining soluble protein content revealed that the average values of OE-17 and OE-19 were higher than those of WT plants, consistent with the proline level (**Figure 9C**). Studies indicated that heat stress could promote the secretion of ROS, and antioxidant enzymes like SOD and POD have ROS-scavenging mechanisms (Wang et al., 2003; Gechev and Petrov, 2020). SOD and POD in transgenic plants were significantly higher than in WT plants following a heat treatment (**Figures 9D, E**). These results suggest that transgenic plants are more tolerant to high-temperature stress than WT plants due to overexpression of *AgHSFa6-1*.

To understand the regulatory function of *AgHSFa6-1* in wild-type and transgenic *Arabidopsis* plants under heat stress, the expressions of *AgHSFa6-1* and seven stress-responsive genes in two lines of transgenic *Arabidopsis* were measured using RT-qPCR **(Figures 9F–N**). Both *AgHSFa6-1* and stress-responsive genes showed similar expression trend in OE-17 and OE-19. The expression levels of seven stress-responsive genes showed that *AtHSP98.7* and *AtAPX1* at all stages of heat stress were higher than those of CK. However, expression levels of *BOB1*, *AtCPN60B*, *AtHSP70-1*, *AtADH2*, and *AtGOLS1* were insignificantly higher than WT during 0-4 h, but expression levels of *AtCPN60B*, *AtHSP70-1*, *AtADH2* were even lower than WT after 12 h. The expression levels of *AtHSP98.7* initially increased between 0 and 4 h, then decreased between 4 and 8 h, and peaked at 24 h. On the contrary, the expression levels of *AtCPN60B* and *AtADH2* were initially decreased and then increased. These genes were significantly upregulated by high-temperature stress and reached their peak expression at 24 h. Moreover, their expression levels were much higher in the transgenic plants than in WT plants. Expression trend of *AgHSFa6-1* in OE-17 and OE-19 was consistent with both transcriptome data and expression analysis in celery, and transcription and translation were relatively faster compared to the other seven genes. This revealed that *AgHSFa6-1* improved the resistance of transgenic *Arabidopsis* to heat stress by positively regulating the expression of abiotic stress-sensitive genes.

## 4 Discussion

Plant HSF TFs are key regulatory network elements and influence the responses to abiotic and biotic stresses. In this study, 29 *AgHSF* genes were identified in celery. HSFs are divided into three classes and could be divided into subgroups based on different protein structures (Kotak et al., 2010). According to the HSF sequences of *Arabidopsis*, the phylogenetic tree formed three distinct classes: A, B, and C. Previous studies revealed that the HSFs belongs to class A were the main regulatory factors during heat stress, whereas the classes B and C members had no transcriptional activity (Takumi et al., 2011; Hu et al., 2015). Numerous studies proved that HSFA2 and HSFA7 are the most heat-sensitive TFs in the *HSF* gene family (Zha et al., 2020); they respond to heat stress by regulating plant hormone induction (Garima et al., 2021), protein synthesis (Wang et al., 2010), and ROS signaling pathway (Chiara et al., 2012). According to the phylogenetic relationships, related genes are always distributed on the same branch of the phylogenetic tree. In this study, phylogenetic analysis revealed that the HSF genes formed 14 subgroups, and the A2, A6, and A7 HSF genes formed an individual branch. The expression level of *AgHSFa6-1* was induced by heat stress, and the transcript abundance trend was similar with *AgHSFa2-2* and *AgHSFa2-3*. However, wether genes of A2 and A6 have similar functions needs further verification. Promoters are one of the most important regulatory elements, determining the temporal and spatial expression of developmentally and physiologically significant genes (Mohsen and Huang, 2022). Several cis-regulatory elements were identified in the *AgHSFa6-1* promoter sequence, especially ARE and STRE elements, that are related to abiotic stress.

High temperature mainly causes the breakdown of the plant’s membrane system, causing abnormal metabolism of water, ions, and organic solutes (Franklin, 2009). Considering that stomata are one of the most critical channels in plants for exchanging carbon dioxide (CO_2_) and water vapors (H_2_O), it is vital to regulate stomatal response to limit plant water loss (Lin et al., 2022; Djemal and Khoudi, 2021). It was reported that stomatal closure is generally triggered *via* the accumulation of ABA and proline (Lim et al., 2015; Maria et al., 2021). Overexpression of *VaHsfC1* in grapes enhances the heat tolerance of transgenic plants by increasing ABA content and decreasing stomatal aperture (Jiao et al., 2022). The present study also revealed that the stomatal apertures of transgenic *Arabidopsis* overexpressing *AgHSFa6-1* under heat stress was significantly lower than that of WT plants, and the proline content was significantly higher than that of WT plants. Previous research demonstrated that proline participated in the ABA metabolism pathway during abiotic stress (Sripinyowanich et al., 2013), indicating that overexpression of the *AgHSFa6-1* gene could enhance thermo-tolerance by promoting proline synthesis and ABA to regulate stomata, although the underlying mechanism remains unknown.

High temperature could increase cellular antioxidant enzyme activity, proline, etc. Plants adapted to a high-temperature environment by improving the process of water metabolism and membrane stability (Liu et al., 2020; Wu et al., 2022). Excessive ROS, particularly H_2_O_2_, is the primary cause of membrane damage, and MDA content is a widely used marker to assess membrane damage under abiotic stress (Zhang et al., 2022). In our research, all the transgenic strains and plants exhibited better thermo-tolerance in physiologically. The transgenic yeast remained alive at 41 °C, but the control yeast stopped growing under 39 °C. Additionally, after 24 h of heat stress, transgenic plants suffered less membrane injury due to the lower level of MDA than the WT plants. High levels of solute protein content and activity of SOD and POD enzymes were detected in transgenic plants, indicating that overexpression of *AgHSFa6-1* could increase the ability of the plant’s ROS-scavenging system to enhance heat tolerance.

Previous studies have revealed that in response to heat stress, the synthesis of certain amino acids (such as serine and proline) and certain protein like HSPs were upregulated, indicating that these synthesis-upregulated amino acids and proteins may be a response to high temperature in plants (Wang et al., 2018; Ren et al., 2019). Numerous studies clarified these functional proteins. Overexpression of the *APX1* gene in *Arabidopsis* can induce the expression of ROS-scavenging related gene for response against abiotic stress (Jiang et al., 2017). *CPN60B* encodes RuBisCO and is involved in photosynthesis (Cheng et al., 2020). BOB1 is a noncanonical heat shock protein that regulates development and heat response, whereas the ABA signal can induce ADH2 (Jurkuta et al., 2009; Nie et al., 2022). Plant HSF transcription factors are downstream components of the signal transduction pathway and maintain regulatory roles for stress-related gene expression (Fragkostfanakis et al., 2015). It was hypothesized that nearly all *AgHSFs* localize to the nucleus so that they may perform transcriptional functions. Expression analysis of seven genes (*AtHSP98.7*, *AtHSP70-1*, *AtBOB1*, *AtCPN60B*, *AtADH2*, *AtAPX1*, and *AtGOLS1*) was carried out to define their interaction with *AgHSFa6-1*. Genes like *AtAPX1* exhibited a sensitive expression profile in response to heat stress compared to WT plants. The expression profiles demonstrated that as the duration of heat stress increased, the expression level of *AgHSFa6-1* increased rapidly in initial heat stress stages, and with heat stress time processing, the expression levels of stress-responsive genes maintained at a high expression level. At the same time, other proteins represented significantly higher expression levels after 12 h of heat stress, suggesting that *AgHSFa6-1* could induce the expression of downstream genes to combat heat stress.

## 5 Conclusion

**Figure 10:**
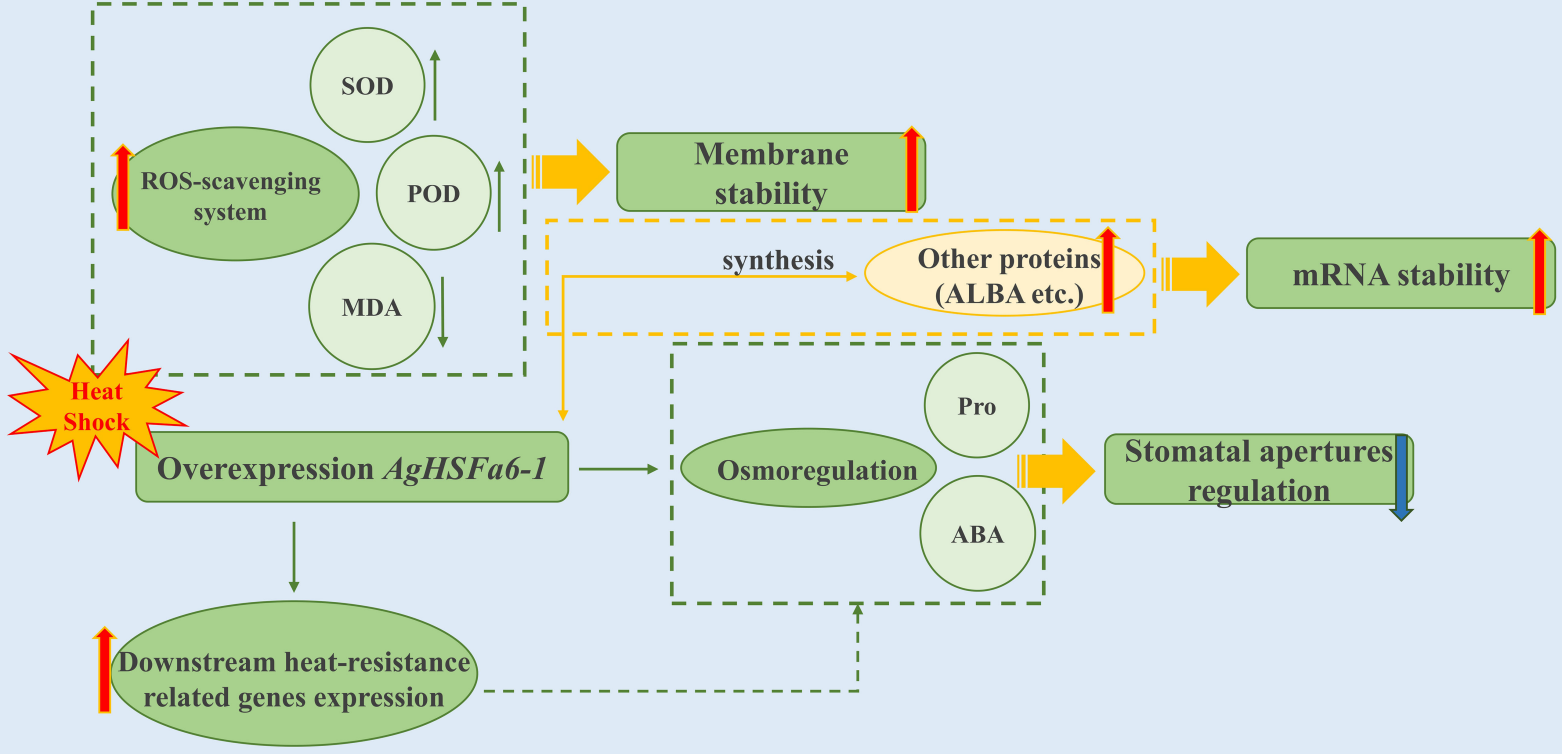
Module-centric mode of the transcriptional regulatory network of *AgHSFa6-1* in response to heat stress.

In summary, we characterized the HSF gene from celery and functional analysis the *AgHSFa6-1* transcription factor. The results revealed that *AgHSFa6-1* acted as a positive regulator of heat tolerance. Cognizant of this, a mode of *AgHSFa6-1* regulation network was established in response to heat stress (**Figure 10**). As plants receive heat shock signals, *AgHSFa6-1* regulates the plant’s resistance to high temperature through three mechanisms. First, overexpression *AgHSFa6-1* induced material like antioxidant enzymes (SOD, POD) to clear excessive ROS, and reduced MDA content to protected membrane from ROS damage, enhancing membrane stability. Second, through osmoregulation, the synthesis of some typical osmotic regulatory substances was upregulated and thus reduced the plant’s stomatal apertures to control water loss. Third, *AgHSFa6-1* promoted the expression of downstream heat-resistance-related genes in plants. Recent research on the HSFA2 and HSFA7 TFs has revealed a novel pathway for plants to enhance thermotolerance, as ALBA protein could bind with HSF mRNAs to maintain stability (Tong et al., 2022). As mentioned earlier, HSFA6 expressed a reasonably close relationship with A2 and A7 HSF TFs. In light of this, *AgHSFa6-1*, as a member of HSFA6, may have the same function. This work classified the AgHSF family and defined the function of *AgHSFa6-1* in response to heat shock. The results above help us understand the molecular mechanisms of celery adaptation to high temperature, and also provide reliable gene resources for molecular breeding of new heat-tolerant cultivars in celery.

## Data availability statement

The original contributions presented in the study are included in the article/**Supplementary Material**. Further inquiries can be directed to the corresponding author.

## Author contributions

Conceptualization, ML, YL, and HT. Methodology, RZ, JZ, JD, XL, and YXL. Resources, YL and XW. Software, YTZ and YZ. Data curation and visualization, YW, QC, and WH. Writing-original draft preparation, ML, RZ, and JZ. Project administration, AX and HT. Funding acquisition, ML and HT. All authors contributed to the article and approved the submitted version.

## Supplementary material

Supplementary Table 1 Primer sequences using in this study.

Supplementary Table 2 Basic information of HSF gene family members in *A. graveolens*.

Supplementary Table 3 Protein sequences of 20 motifs in AgHSFs.

## References

[B1] Bates L. S. Waldren R. P. Teare I. D. (1973). Rapid determination of free proline for water-stress studies. Plant Soil. 39, 205–207. doi: 10.1007/BF00018060

[B2] Cao K. Yu J. Xu D. W. Ai K. Q. Bao E. C. Zou Z. R. (2018). Exposure to lower red to far-red light ratios improve tomato tolerance to salt stress. BMC Plant Biol. 18 (1), 92. doi: 10.1186/s12870-018-1310-9 29793435 PMC5968587

[B3] Chan-Schaminet K. Y. Baniwal S. K. Bublak D. Nover L. Scharf K. D. (2009). Specific interaction between tomato HsfA1 and HsfA2 creates hetero-oligomeric superactivator complexes for synergistic activation of heat stress gene expression. J. Biol. Chem. 284 (31), 20848–20857. doi: 10.1074/jbc.M109.007336 19491106 PMC2742850

[B4] Chen C. Chen H. Zhang Y. Thomas H. R. Frank M. G. He Y.. (2020). TBtools: an integrative toolkit developed for interactive analyses of big biological data. Mol. Plant 13 (8), 1194–1202. doi: 10.1016/j.molp.2020.06.009 32585190

[B5] Chen S. S. Jiang J. Han X. J. Zhang Y. X. Zhuo R. Y. (2018). Identification, expression analysis of the hsf family, and characterization of class A4 in *Sedum Alfredii* hance under cadmium stress. Int. J. Mol. Sci. 19 (4), 1216. doi: 10.3390/ijms19041216 29673186 PMC5979518

[B6] Cheng X. Q. Fang T. Y. Zhao E. H. Zheng B. G. Huang B. R. An Y.. (2020). Protective roles of salicylic acid in maintaining integrity and functions of photosynthetic photosystems for alfalfa *(Medicago sativa* l.) tolerance to aluminum toxicity. Plant Physiol. Biochem. 155, 570–578. doi: 10.1016/j.plaphy.2020.08.028 32846392

[B7] Chiara P. Valeria B. Pierdomenico P. (2012). ROS signaling as common element in low oxygen and heat stress. Plant Physiol. Biochem. 59, 3–10. doi: 10.1016/j.plaphy.2012.02.016 22417734

[B8] Djemal R. Khoudi H. (2021). The barley SHN1-type transcription factor HvSHN1 imparts heat, drought and salt tolerances in transgenic tobacco. Plant Physiol. Biochem. 164, 44–53. doi: 10.1016/j.plaphy.2021.04.018 33962230

[B9] Fan K. Mao Z. J. Ye F. T. Pan X. F. Li Z. W. Lin W. W.. (2021). Genome-wide identification and molecular evolution analysis of the heat shock transcription factor (HSF) gene family in four diploid and two allopolyploid *Gossypium* species. Genomics. 113 (5), 3112–3127. doi: 10.1016/j.ygeno.2021.07.008 34246694

[B10] Fragkostefanakis S. Mesihovic A. Simm S. Paupière M. J. Hu Y. Paul P.. (2016). HsfA2 controls the activity of developmentally and stress-regulated heat stress protection mechanisms in tomato male reproductive tissues. Plant Physiol. 170 (4), 2461–2477. doi: 10.1104/pp.15.01913 26917685 PMC4825147

[B11] Fragkostfanakis S. Roth S. Schleiff E. Scharf K. D. (2015). Prospects of engineering thermotolerance in crops through modulation of heat stress transcription factor and heat shock protein networks. Plant Cell Environ. 38 (9), 1881–1895. doi: 10.1111/pce.12396 24995670

[B12] Franklin K. A. (2009). Light and temperature signal crosstalk in plant development. Curr. Opin. Plant Biol. 12 (1), 63–68. doi: 10.1016/j.pbi.2008.09.007 18951837

[B13] Fu Y. L. Ma H. L. Chen S. Y. Gu T. Y. Gong J. M. (2018). Control of proline accumulation under drought *via* a novel pathway comprising the histone methylase CAU1 and the transcription factor ANAC055. J. Exp. Bot. 69 (3), 579–588. doi: 10.1093/jxb/erx419 29253181 PMC5853435

[B14] Garima S. Neelam K. S. Anil G. (2021). Tango between ethylene and HSFA2 settles heat tolerance. Trends Plant Sci. 26 (5), 429–432. doi: 10.1016/j.tplants.2021.03.003 33744161

[B15] Gechev T. Petrov V. (2020). Reactive oxygen species and abiotic stress in plants. Int. J. Mol. Sci. 21 (20), 7433. doi: 10.3390/ijms21207433 33050128 PMC7588003

[B16] Guo M. Liu J. H. Ma X. Luo D. X. Gong Z. H. Lu M. H. (2016). The plant heat stress transcription factors (HSFs): structure, regulation, and function in response to abiotic stresses. Front. Plant Sci. 7. doi: 10.3389/fpls.2016.00114 PMC4746267 26904076

[B17] Hu Y. Han Y. T. Wei W. Li Y. J. Zhang K. Gao Y. R.. (2015). Identification, isolation, and expression analysis of heat shock transcription factors in the diploid woodland strawberry fragaria vesca. Front. Plant Sci. 6. doi: 10.3389/fpls.2015.00736 PMC4569975 26442049

[B18] Hu R. B. Qi G. Kong Y. Z. Kong D. J. Gao Q. Zhou G. K. (2010). Comprehensive analysis of NAC domain transcription factor gene family in *Populus trichocarpa* . BMC Plant Biol. 10, 145. doi: 10.1186/1471-2229-10-145 20630103 PMC3017804

[B19] Jacob P. Hirt H. Bendahmane A. (2017). The heat-shock protein/chaperone network and multiple stress resistance. Plant Biotechnol. J. 15, 405–414. doi: 10.1111/pbi.12659 27860233 PMC5362687

[B20] Jiang L. Y. Hu W. J. Qian Y. X. Ren Q. Y. Zhang J. (2021). Genome-wide identification, classification and expression analysis of the hsf and Hsp70 gene families in maize. Gene. 770, 145348. doi: 10.1016/j.gene.2020.145348 33333230

[B21] Jiang L. Wang W. Y. Chen Z. P. Gao Q. C. Xu Q. X. Cao H. M. (2017). A role for APX1 gene in lead tolerance in *Arabidopsis thaliana* . Plant Sci. 256, 94–102. doi: 10.1016/j.plantsci.2016.11.015 28167043

[B22] Jiao S. Z. Guo C. Yao W. K. Zhang N. B. Zhang J. Y. Xu W. R. (2022). An amur grape *VaHsfC1* is involved in multiple abiotic stresses. Sci. Hortic. 295, 110785. doi: 10.1016/j.scienta.2021.110785

[B23] Jurkuta R. J. Kaplinsky N. J. Spindel J. E. Barton M. K. (2009). Partitioning the apical domain of the *Arabidopsis* embryo requires the BOBBER1 NudC domain protein. Plant Cell. 21 (7), 1957–1971. doi: 10.1105/tpc.108.065284 19648297 PMC2729608

[B24] Kotak S. Port M. A. Bicker F. Pascal K. D. (2010). Characterization of c-terminal domains of *Arabidopsis* heat stress transcription factors (Hsfs) and identification of a new signature combination of plant class a hsfs with AHA and NES motifs essential for activator function and intracellular localization. Plant J. 39 (1), 98–112. doi: 10.1111/j.1365-313X.2004.02111.x 15200645

[B25] Kumar A. Changwal C. Thapa B. Tanpure R. S. Hada A. Singh P. K.. (2021). Transcription factors: a tool box for countering the effect of abiotic stresses. Stress Tolerance Hort Crops., 169–192. doi: 10.1016/B978-0-12-822849-4.00019-X

[B26] Lescot M. Dehais P. Thijs G. Marchal K. Moreau Y. Van de Peer Y.. (2002). PlantCARE, a database of plant *cis*-acting regulatory elements and a portal to tools for in silico analysis of promoter sequences. Nucleic Acids Res. 30 (1), 325–327. doi: 10.1093/nar/30.1.325 11752327 PMC99092

[B27] Li D. L. He Y. J. Li S. H. Shi S. L. Li L. Z. Liu Y.. (2021). Geneme-wide characterization and expression analysis of *AP2/ERF* genes in eggplant *(solanum melongena* l.). Plant Physiol. Biochem. 167, 492–503. doi: 10.1016/j.plaphy.2021.08.006 34425394

[B28] Li M. Y. Hou X. L. Wang F. Tan G. F. Xu Z. S. Xiong A. S. (2018). Advances in the research of celery, an important apiaceae vegetable crop. Crit. Rev. Biotechnol. 38 (2), 172–183. doi: 10.1080/07388551.2017.1312275 28423952

[B29] Li T. Huang Y. Khadr A. Wang Y. H. Xu Z. S. Xiong A. S. (2020). *DcDREB1A*, a DREB-binding transcription factor from *Daucus carota*, enhances drought tolerance in transgenic *Arabidopsis thaliana* and modulates lignin levels by regulating lignin-biosynthesis-related genes. Environ. Exp. Bot. 169, 103896. doi: 10.1016/j.envexpbot.2019.103896

[B30] Li M. Y. Li J. Zhang R. Lin Y. X. Xiong A. S. Tan G. F.. (2022a). Combined analysis of the metabolome and transcriptome to explore heat stress responses and adaptation mechanisms in celery *(Apium graveolens *L.). Int. J. Mol. Sci. 23 (6), 3367. doi: 10.3390/ijms23063367 35328788 PMC8950972

[B31] Li M. Y. Li X. Y. Zhou J. Sun Y. Du J. G. Wang Z.. (2022b). Genome-wide identification and analysis of terpene synthase *(TPS*) genes in celery reveals their regulatory roles in terpenoid biosynthesis. Front. Plant Sci. 13. doi: 10.3389/fpls.2022.1010780 PMC9557977 36247575

[B32] Li M. Y. Wang F. Jiang Q. Ma J. Xiong A. S. (2014). Identification of SSRs and differentially expressed genes in two cultivars of celery (*Apium graveolens *L.) by deep transcriptome sequencing. Hortic. Res. 1, 10. doi: 10.1038/hortres.2014.10 26504532 PMC4596314

[B33] Li G. L. Zhang H. N. Shao H. Wang G. Y. Zhang Y. Y. Zhang Y. J.. (2019). *ZmHsf05*, A new heat shock transcription factor from *Zea mays *L. improves thermotolerance in *Arabidopsis thaliana* and rescues thermotolerance defectes of the *athsfa2* mutant. Plant Sci. 284, 375–384. doi: 10.1016/j.plantsci.2019.03.002 31128708

[B34] Lim C. W. Baek W. Jung J. Kim J. H. Lee S. C. (2015). Function of ABA in stomatal defense against biotic and drought stresses. Int. J. Mol. Sci. 16 (7), 15251–15270. doi: 10.3390/ijms160715251 26154766 PMC4519898

[B35] Lin A. P. Chen Y. T. Ponce G. Acevedo F. E. Anderson C. T. Ali J. G.. (2022). Stomata-mediated interactions between plants, herbivores, and the environment. Trends Plant Sci. 27 (3), 287–300. doi: 10.1016/j.tplants.2021.08.017 34580024

[B36] Liu G. T. Chai F. M. Wang Y. Jiang J. Z. Duan W. Wang Y. T.. (2018). Genome-wide identification and classification of hsf family in grape, and their transcriptional analysis under heat acclimation and heat stress. Hortic. Plant J. 4 (4), 133–143. doi: 10.1016/j.hpj.2018.06.001

[B37] Liu Z. L. Li L. Luo Z. S. Zeng F. F. Jiang L. Tang K. C. (2016). Effect of brassinolide on energy status and proline metabolism in postharvest bamboo shoot during chilling stress. Postharvest Biol. Technol. 111, 240–246. doi: 10.1016/j.postharvbio.2015.09.016

[B38] Liu X. S. Liang C. C. Hou S. G. Wang X. Chen D. H. Shen J. L.. (2020). The LRR-RLK protein HSL3 regulates stomatal closure and the drought stress response by modulating hydrogen peroxide homeostasis. Front. Plant Sci. 11. doi: 10.3389/fpls.2020.548034 PMC7728693 33329622

[B39] Macadam J. W. Nelson C. J. Sharp R. E. (1992). Peroxidase activity in the leaf elongation zone of tall fescue: i. spatial distribution of ionically bound peroxidase activity in genotypes differing in length of the elongation zone. Plant Physiol. 99 (3), 879–885. doi: 10.1104/pp.99.3.879 16669015 PMC1080559

[B40] Maria L. D. Christian J. S. Teresa C. M. Vincente M. Barbara B. U. Rosa M. R. (2021). Synchronization of proline, ascorbate and oxidative stress pathways under the combination of salinity and heat in tomato plants. Environ. Exp. Bot. 183, 104351. doi: 10.1016/j.envexpbot.2020.104351

[B41] Meiri D. Tazat K. Cohen-Peer R. Farchi-Pisanty O. Aviezer-Hagai K. Avni A.. (2010). Involvement of *Arabidopsis* ROF2 (FKBP65) in thermotolerance. Plant Mol. Biol. 72 (1-2), 191–203. doi: 10.1007/s11103-009-9561-3 19876748

[B42] Mohsen H. Huang S. S. (2022). Elucidating the biology of transcription factor-DNA interaction for accurate identification of cis-regulatory elements. Curr. Opin. Plant Biol. 68, 102232. doi: 10.1016/j.pbi.2022.102232 35679803 PMC10103634

[B43] Nie X. Z. Mohammed M. Abir U. I. Robert D. H. Claudio S. (2022). Anaerobiosis modulation of two phytoglobins in barley *(Hordeum vulgare *L.), and their regulation by gibberellin and abscisic acid in aleurone cells. Plant Physiol. Biochem. 182, 174–181. doi: 10.1016/j.plaphy.2022.04.014 35504225

[B44] Nover L. Bharti K. Doring P. Mishra S. Ganguli A. Scharf K. (2001). *Arabidopsis* And the heat stress transcription factor world: how many heat stress transcription factors do we need. Cell Stress Chaperones. 6 (3), 177–189. doi: 10.1379/1466-1268(2001)006<0177:aathst>2.0.co;2 11599559 PMC434399

[B45] Pfaffla M. W. (2001). A new mathematical model for relative quantification in real-time RT-PCR. Nucleic Acids Res. 29 (9), e45. doi: 10.1093/nar/29.9.e45 11328886 PMC55695

[B46] Qian J. Chen J. Liu Y. F. Yang L. L. Li W. P. Zhang L. M. (2014). Overexpression of *Arabidopsis HsfA1a* enhances diverse stress tolerance by promoting stress-induced hsp expression. Genet. Mol. Res. 13 (1), 1233–1243. doi: 10.4238/2014.february.27.8 24634180

[B47] Ren S. X. Ma K. B. Lu X. G. Chen G. Cui J. W. Tong P. X.. (2019). Transcriptomic and metabolomic analysis of the heat-stress response of *Populus tomentosa* Carr. Forests. 10 (5), 383. doi: 10.3390/f10050383

[B48] Richard N. R. (2004). Celery diseases and their management. Dis. fruits vegetables. 1, 441–453. doi: 10.1007/1-4020-2606-4_11

[B49] Shannon P. Markiel A. Ozier O. Baliga N. S. Wang J. T. Ramage D.. (2003). Cytoscape: a software environment for integrated models of biomolecular interaction networks. Genome Res. 13 (11), 2498–2504. doi: 10.1101/gr.1239303 14597658 PMC403769

[B50] Sripinyowanich S. Klomsakul P. Boonburapong B. Bangyeekhun T. Asami T. Gu H.. (2013). Exogenous ABA induces salt tolerance in indica rice (*Oryza sativa *L.): the role of *OsP5CS1* and *OsP5CR* gene expression during salt stress. Environ. Exp. Bot. 86, 94–105. doi: 10.1016/j.envexpbot.2010.01.009

[B51] Takumi Y. Naohiko O. Jun N. Satoshi K. Junya M. Kazuo N.. (2011). *Arabidopsis HsfA1* Transcription factors function as the main positive regulators in heat shock-responsive gene expression. Mol. Genet. Genom. 286 (5-6), 321–332. doi: 10.1007/s00438-011-0647-7 21931939

[B52] Tan B. Yan L. Li H. N. Lian X. D. Cheng J. Wang W.. (2021). Genome-wide identification of HSF family in peach and functional analysis of *PpHSF5* involvement in root and aerial organ development. PeerJ. 9, e10961. doi: 10.7717/peerj.10961 33763299 PMC7958895

[B53] Tong J. J. Ren Z. T. Sun L. H. Zhou S. X. Yuan W. Hui Y. F.. (2022). ALBA proteins confer thermotolerance through stabilizing HSF messenger RNAs in cytoplasmic granules. Nat. Plants. 8 (7), 778–791. doi: 10.1038/s41477-022-01175-1 35817823

[B54] Waadt R. Seller C. A. Hsu P. K. Takahashi Y. Munemasa S. Schroeder J. I. (2022). Plant hormone regulation of abiotic stress responses. Nat. Rev. Mol. Cell Biol. 23 (10), 680–694. doi: 10.1038/s41580-022-00479-6 35513717 PMC9592120

[B55] Wang L. J. Fan L. Loescher W. Duan W. Liu G. L. Cheng J. S.. (2010). Salicylic acid alleviates decreases in photosynthesis under heat stress and accelerates recovery in grapevine leaves. BMC Plant Biol. 10, 34. doi: 10.1186/1471-2229-10-34 20178597 PMC2848757

[B56] Wang J. Q. Hasegawa T. Li L. Q. Lam S. K. Zhang X. H. Liu X. Y.. (2018). Changes in grain protein and amino acids composition of wheat and rice under short-term increased [CO_2_] and temperature of canopy air in a paddy from East China. New Phytol. 222 (2), 726–734. doi: 10.1111/nph.15661 30586149

[B57] Wang R. Mao C. J. Jiang C. H. Zhang L. Peng S. Y. Zhang Y.. (2021). One heat shock transcription factor confers high thermal tolerance in clematis plants. Int. J. Mol. Sci. 22 (6), 2900. doi: 10.3390/ijms22062900 33809330 PMC7998627

[B58] Wang W. S. Rong Y. Wang X. W. Wang C. Z. Zhang C. L. Huo Z. L.. (2022). Estimating sunflower canopy conductance under the influence of soil salinity. Agric. For Meteorol. 314 (4), 108778. doi: 10.1016/j.agrformet.2021.108778

[B59] Wang W. X. Vinocur B. Altman A. (2003). Plant responses to drought, salinity and extreme temperatures: towards genetic engineering for stress tolerance. Planta. 218 (1), 1–14. doi: 10.1007/s00425-003-1105-5 14513379

[B60] Wu J. T. Gao T. Hu J. N. Zhao L. Yu C. Ma F. (2022). Research advances in function and regulation mechanisms of plant small heat shock proteins (sHSPs) under environmental stresses. Sci. Total Environ. 825, 154054. doi: 10.1016/j.scitotenv.2022.154054 35202686

[B61] Zha Q. Xi X. J. He Y. N. Jiang A. L. (2020). Transcriptomic analysis of the leaves of two grapevine cultivars under high-temperature stress. Sci. Hortic. 265 (4), 109265. doi: 10.1016/j.scienta.2020.109265

[B62] Zhang X. Henriques R. Lin S. S. Niu Q. W. Chua N. H. (2006). Agrobacterium-mediated transformation of *Arabidopsis thaliana* using the floral dip method. Nat. Protoc. 1 (2), 641–646. doi: 10.1038/nprot.2006.97 17406292

[B63] Zhang X. Y. Zhou Y. Li J. Gu X. Y. Zhao L. N. Li B.. (2022). Pichia caribbica improves disease resistance of cherry tomatoes by regulating ROS metabolism. Biol. Control. 169 (5), 104870. doi: 10.1016/j.biocontrol.2022.104870

[B64] Zhao X. G. Sui X. Y. Zhao L. X. Gao X. X. Wang J. X. Wen X. Z.. (2022). Morphological and physiological response mechanism of lettuce *(Lactuca Sativa *L.) to consecutive heat stress. Sci. Hortic. 301, 111112. doi: 10.1016/j.scienta.2022.111112

[B65] Zhou M. Zheng S. G. Liu R. Lu J. Lu L. Zhang C. H.. (2019). Genome-wide identification, phylogenetic and expression analysis of the heat shock transcription factor family in bread wheat *(Triticum aestivum *L.). BMC Genom. 20 (1), 505. doi: 10.1186/s12864-019-5876-x PMC6580518 31215411

