# Supplementary material for: Comprehensive analysis of HSF genes from celery (Apium graveolens L.) and functional characterization of AgHSFa6-1 in response to heat stress
Source: Front Plant Sci. 2023 May 8;14:1132307. doi: 10.3389/fpls.2023.1132307 (PMC10202177; doi:10.3389/fpls.2023.1132307)
Supplement: Supplementary Table 1 — Primer sequences using in this study. [file Table_1.docx]

Table S1 Primer sequences using in this study

| Gene ID | Gene name | Forward primer (5’-3’) | Reverse primer (5’-3’) | Primer use |
| --- | --- | --- | --- | --- |
| *Ag11G04087.1* | *AgHSFa6-1* | ATGGAGCAAAAGCTACAAGG | CTAATTTGGGGTTGAACCTAAG | Gene cloning |
| *Ag11G04087.1* | *AgHSFa6-1* | ACGGGGGACTCTAGAGGATCCATGGAGCAAAAGCTACAAGG | GCCCTTGCTCACCATGGATCCCTAATTTGGGGTTGAACCTAAG | Subcellular localization |
| *Ag11G04087.1* | *AgHSFa6-1* | GGATCCATGATGGCAGCATCTTTA | CTTAAGCTAATTTGGGGTTGAACCTAAG | Transformation in yeast |
| *Ag11G04087.1* | *AgHSFa6-1* | TTTACAATTACCATGGGATCCATGGAGCAAAAGCTACAAGG | ACCGATGATACGAACGAGCTCCTAATTTGGGGTTGAACCTAAG | Transformation in plant |
| *Ag11G04087.1* | *AgHSFa6-1* | GGATTCGGTAAGCAAGGAGTGAG | ATGAGCCAGGACACTGACATCTTC | RT-qPCR |
| *Ag9G02596.1* | *AgHSFa2-1* | CCGACAACAACAGGAGGAGTCC | AGCGAGCCTTCTCTTCCTTCCA | RT-qPCR |
| *Ag7G00386.1* | *AgHSFa2-2* | AGGCAACAGAGGCTAAGCAACA | GCGGCTGACAAGAATGACTCCA | RT-qPCR |
| *Ag6G01277.1* | *AgHSFa2-3* | CAAGTGGCTGGCGTCTGTGT | TCGACTCCAGGTGTTGCTGTCT | RT-qPCR |
| *Ag9G02114.1* | *AgHSFa6-2* | GCAGGGATTCGGTAGAGCAAGG | AGCCAGGACACTGACATCTTCC | RT-qPCR |
| *Ag5G00279.1* | *AgHSFb1-1* | TCAACCTCCACCACCTCATCCC | CCACCTTCATTCGCCGAGTCAA | RT-qPCR |
| *Ag4G01086.1* | *AgHSFb1-2* | ATACTCCAGTCGTGTGGCAGGT | TCAGGCACTATCTTGCGGAAGC | RT-qPCR |
| *Ag3G02367.1* | *AgHSFb2-1* | GCGAGAACGAGCGGTTGAGAA | CCTGCTGCTGTTGCGAACTACT | RT-qPCR |
| *Ag2G01987.1* | *AgHSFb2-2* | CCGCTTGATCTGATGCCGTTGA | TCCAGCCCACCGCTTATCTTGA | RT-qPCR |
| *Ag9G02441* | *AgTUB* | TGGTGGCACTGGATCTGGTATGG | ACTTTCGGAGAAGGGAAGACTGA | RT-qPCR |
| *AT2G25140* | *AtHSP98.7* | TGCTTGGACGAGGTGAACTGAG | CGTTCGGTGATGTAGCGGTCTG | RT-qPCR |
| *AT5G53400* | *AtBOB1* | AAGCAGTAGCAGCCGACCCAT | AACTTCTCCTTCGCCGCCCTAA | RT-qPCR |
| *AT5G02500* | *AtHSP70-1* | TGCGTGAGATTGCTGAGGCTTA | CGGCTGTAGGCTCGTTGATGAT | RT-qPCR |
| *AT1G55490* | *AtCPN60B* | AGCAAGCGGAGCAAGATTACGA | TGGAAGCAAGACGAAGCAGAGT | RT-qPCR |
| *AT5G43940* | *AtADH2* | GCCGAACAAGCCTCTGGTCATC | CCAAGTGTAAGCGTCGGTGTGA | RT-qPCR |
| *AT1G07890* | *AtAPX1* | ACTACCCAACCGTGAGCGAAGA | TGCCATGCGAGTCGGACCAT | RT-qPCR |
| *AT2G47180* | *AtGOLS1* | ATGGAGTCACACGCCGCAATAC | TGACGCCATAACATCGCAAGGA | RT-qPCR |
| *AT3G18780* | *AtACT2* | GAAATCACAGCACTTGCACC | AAGCCTTTGATCTTGAGAGC | RT-qPCR |
