# Supplementary material for: Comprehensive analysis of HSF genes from celery (Apium graveolens L.) and functional characterization of AgHSFa6-1 in response to heat stress
Source: Front Plant Sci. 2023 May 8;14:1132307. doi: 10.3389/fpls.2023.1132307 (PMC10202177; doi:10.3389/fpls.2023.1132307)
Supplement: Supplementary Table 2 — Basic information of HSF gene family members in A. graveolens. [file Table_2.docx]

- Table S2 Basic information of HSF gene family members in *A. graveolens*

| Name | Locus Id | Size (aa) | Aliphatic I,L,V | Aromatics F,W,Y | positive amino acids (%) | negative amino acids (%) | pI | Molecular weight (Da) | Subcellular localization | Protein GRAVY |
| --- | --- | --- | --- | --- | --- | --- | --- | --- | --- | --- |
| AgHSFa1-1 | Ag10G01924.1 | 284 | 60 | 25 | 12 | 14 | 5.91 | 32543.86 | nucl/nucl | -0.573 |
| AgHSFa1-2 | Ag2G02167.1 | 487 | 93 | 30 | 10 | 13 | 5.72 | 54582.90 | nucl/nucl | -0.697 |
| AgHSFa1-3 | Ag6G00810.1 | 503 | 94 | 31 | 9 | 13 | 4.97 | 55586.82 | nucl/nucl | -0.609 |
| AgHSFa2-1 | Ag9G02596.1 | 375 | 69 | 31 | 13 | 16 | 5.38 | 42993.54 | nucl/nucl | -0.674 |
| AgHSFa2-2 | Ag7G00386.1 | 393 | 68 | 29 | 11 | 15 | 5.28 | 44428.55 | nucl/nucl | -0.750 |
| AgHSFa2-3 | Ag6G01277.1 | 357 | 63 | 28 | 10 | 15 | 4.90 | 40556.53 | nucl/nucl | -0.663 |
| AgHSFa3-1 | Agr01383 | 432 | 88 | 37 | 10 | 14 | 5.23 | 48642.96 | nucl/nucl | -0.452 |
| AgHSFa4-1 | Agr07325 | 402 | 70 | 34 | 12 | 15 | 5.37 | 45917.94 | nucl/nucl | -0.777 |
| AgHSFa4-2 | Agr16348 | 406 | 76 | 31 | 11 | 15 | 5.27 | 46151.19 | nucl/nucl | -0.742 |
| AgHSFa4-3 | Agr26377 | 312 | 60 | 24 | 12 | 15 | 5.39 | 36001.16 | nucl/nucl | -0.773 |
| AgHSFa5-1 | Ag11G03470.1 | 440 | 82 | 34 | 11 | 12 | 6.25 | 49879.48 | nucl/nucl | -0.631 |
| AgHSFa5-2 | Ag8G02137.1 | 478 | 78 | 37 | 12 | 14 | 5.56 | 53905.49 | nucl/nucl | -0.826 |
| AgHSFa5-3 | Ag3G00561.1 | 441 | 75 | 38 | 9 | 15 | 5.19 | 50272.60 | nucl/nucl | -0.751 |
| AgHSFa6-1 | Ag11G04087.1 | 343 | 61 | 33 | 13 | 15 | 6.09 | 39518.55 | nucl/nucl | -0.776 |
| AgHSFa6-2 | Ag9G02114.1 | 353 | 61 | 31 | 12 | 16 | 5.08 | 40222.06 | nucl/nucl | -0.793 |
| AgHSFa7-1 | Ag9G01747.1 | 328 | 69 | 22 | 12 | 14 | 5.96 | 36894.73 | nucl/nucl | -0.577 |
| AgHSFa8-1 | AgUnG01018.1 | 370 | 79 | 30 | 12 | 17 | 4.83 | 42628.45 | chlo/nucl | -0.568 |
| AgHSFa9-1 | Ag8G00253.1 | 384 | 70 | 28 | 13 | 17 | 5.16 | 44166.86 | nucl/cyto | -0.718 |
| AgHSFa9-2 | Ag7G00674.1 | 232 | 44 | 18 | 10 | 15 | 4.88 | 26450.87 | cyto_nucl/cyto_nucl | -0.556 |
| AgHSFa9-3 | Ag5G02653.1 | 339 | 69 | 31 | 11 | 16 | 4.93 | 39160.98 | cyto/C | -0.646 |
| AgHSFb1-1 | Ag5G00279.1 | 281 | 49 | 23 | 15 | 14 | 8.57 | 31451.43 | nucl/nucl | -0.698 |
| AgHSFb1-2 | Ag4G01086.1 | 282 | 47 | 25 | 15 | 14 | 8.55 | 31837.73 | nucl/nucl | -0.781 |
| AgHSFb2-1 | Ag3G02367.1 | 312 | 54 | 20 | 11 | 13 | 5.15 | 34525.28 | nucl/nucl | -0.729 |
| AgHSFb2-2 | Ag2G01987.1 | 305 | 50 | 22 | 11 | 15 | 5.02 | 34140.72 | nucl/nucl | -0.792 |
| AgHSFb3-1 | Ag4G01404.1 | 246 | 39 | 20 | 16 | 13 | 9.31 | 28160.12 | nucl/nucl | -0.746 |
| AgHSFb3-2 | Ag10G02349.1 | 248 | 42 | 22 | 16 | 14 | 8.88 | 28649.52 | nucl/nucl | -0.824 |
| AgHSFb4-1 | Ag2G00355.1 | 336 | 60 | 30 | 9 | 9 | 7.28 | 38458.36 | nucl/nucl | -0.679 |
| AgHSFb4-2 | Ag10G01872.1 | 320 | 57 | 33 | 10 | 9 | 8.44 | 36350.95 | nucl/nucl | -0.595 |
| AgHSFc1-1 | Ag10G01542.1 | 313 | 55 | 29 | 12 | 14 | 5.17 | 35419.89 | nucl/nucl | -0.541 |

Note: cyto: cytoplasm; chlo: chloroplast; C: chloroplast.

The gene sequences can be downloaded with the Locus Id in celery genome database (<http://apiaceae.njau.edu.cn/celerydb>) and BIO2DB (<http://celerydb.bio2db.com/>).
