# Supplementary material for: Comprehensive analysis of HSF genes from celery (Apium graveolens L.) and functional characterization of AgHSFa6-1 in response to heat stress
Source: Front Plant Sci. 2023 May 8;14:1132307. doi: 10.3389/fpls.2023.1132307 (PMC10202177; doi:10.3389/fpls.2023.1132307)
Supplement: Supplementary Table 3 — Protein sequences of 20 motifs in AgHSFs. [file Table_3.docx]

**Table S3 Protein sequences of 20 motifs in AgHSFs**

| Motif | *E*-value | Width | Best Pssible Match |
| --- | --- | --- | --- |
| Motif 1 | 5.4e-793 | 39 | DLLPKYFKHNNFSSFVRQLNTYGFRKVDPDRWEFANEGF |
| Motif 2 | 1.7e-387 | 38 | PPFLTKTYDMVDDPATDHIVSWSSDGNSFVVWBPPEFA |
| Motif 3 | 1.2e-226 | 50 | LKRDKNMLMLELVKLRQZQQNTKNQLLAMEZRJQGMEQRQQQMMSFLAKA |
| Motif 4 | 3.3e-191 | 16 | JRGZKHLLKNIHRRKP |
| Motif 5 | 8.0e-060 | 46 | SSSSSYTALVEENERLKKENEQLSSELAQMKKLCNEJIVMVSNYVK |
| Motif 6 | 3.0e-069 | 35 | APAPFLTKTYZMVDDPTTDSIVSWSHNGRSFIVWD |
| Motif 7 | 7.1e-02 | 13 | GVNDVFWEZLLTE |
| Motif 8 | 5.9e-022 | 24 | EGPKLFGVPLQNKKKKRKREEIIG |
| Motif 9 | 2.3e-021 | 15 | CVELGRYGIEEEIER |
| Motif 10 | 2.8e-017 | 12 | PQPMEGLHEVGP |
| Motif 11 | 4.1e-0.17 | 15 | GNRKRRLPRSSSLQD |
| Motif 12 | 2.1e-010 | 29 | KSTSGFNSELLERLESSLTFWENVLHDVA |
| Motif 13 | 7.7e-005 | 13 | VHSHSAQNLHGQG |
| Motif 14 | 2.0e-002 | 25 | GDYYGLPVSELDILALEIQGFGRAR |
| Motif 15 | 8.5e-003 | 22 | DFDQSTSIADSPAISYIYJBIE |
| Motif 16 | 2.6e-002 | 19 | WWDIENLQNLTEQMGHLRP |
| Motif 17 | 1.4e-002 | 19 | ISPTDSGDZVNNWSCSSPP |
| Motif 18 | 4.7e-002 | 32 | VVQQKGFNGQQFGNGCDQQDYNHQLNNITKQ |
| Motif 19 | 1.4e-001 | 44 | DQASDYSRQQQEKSADAETDIESFLLAAVDNESTDNINSPKVPT |
| Motif 20 | 4.6e-001 | 21 | KPRFVLEKDDLGLNLMPPSPC |
