# Supplementary figures and images for: Comprehensive analysis of HSF genes from celery (Apium graveolens L.) and functional characterization of AgHSFa6-1 in response to heat stress
Source: Front Plant Sci. 2023 May 8;14:1132307. doi: 10.3389/fpls.2023.1132307 (PMC10202177; doi:10.3389/fpls.2023.1132307)

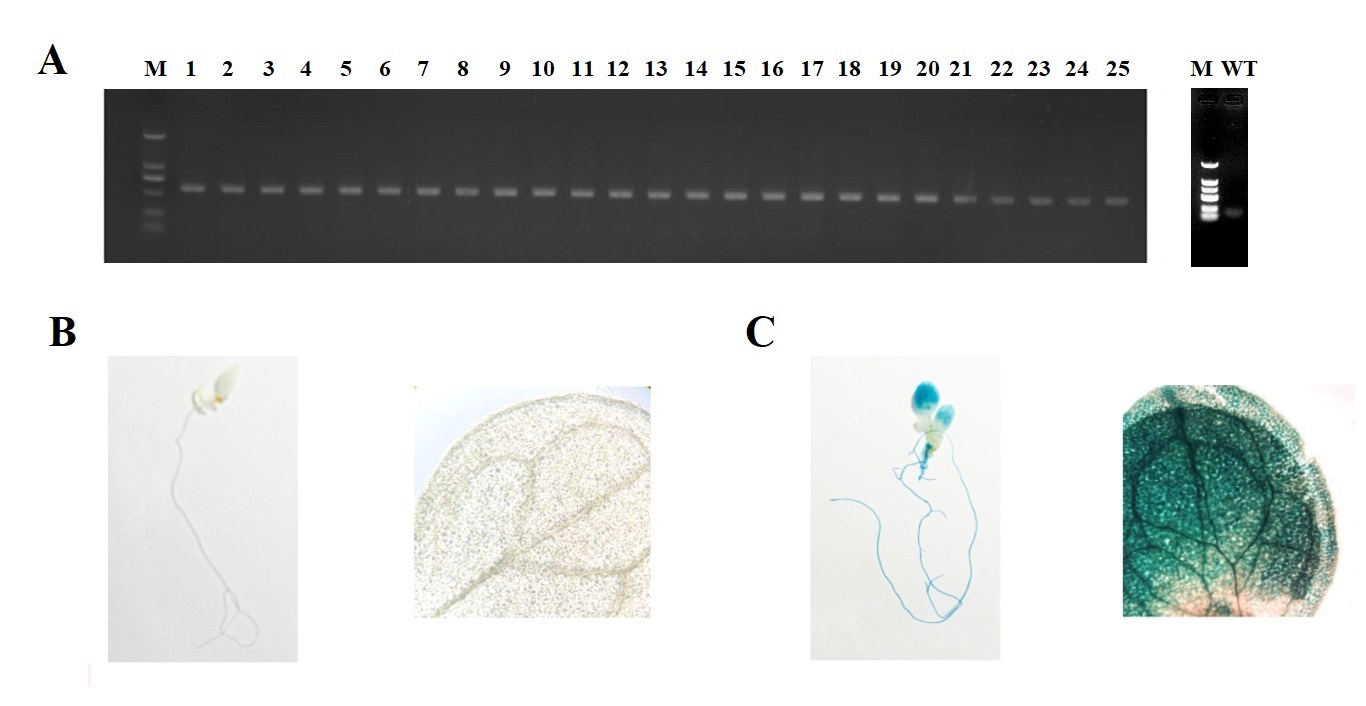

Supplement: Supplementary file 4 [file Image_1.jpeg]
